# Hip Capsular Closure in Distraction: A Technique to Allow Easier Closure of T and Interportal Capsulotomies

**DOI:** 10.1016/j.eats.2023.03.023

**Published:** 2023-07-10

**Authors:** Emre Anil Özbek, Liane Miller, Michael James, Craig S. Mauro

**Affiliations:** a Department of Orthopedic Surgery, University of Pittsburgh Medical Center, Pittsburgh, Pennsylvania, U.S.A.; b Department of Orthopedics and Traumatology, Ankara University, Ankara, Turkey; c Burke and Bradley Orthopedics, Pittsburgh, Pennsylvania, U.S.A.

## Abstract

Capsule closure during hip arthroscopy is increasingly being shown to optimize outcomes and minimize complications. Although various techniques and suture configurations have been described, closure of the hip capsule remains a technically challenging step for many hip arthroscopists. The purpose of this Technical Note is to summarize capsular management in arthroscopic hip-preservation surgery and to outline a technique of passing capsule sutures under hip traction. This technique is useful, as it facilitates adequate visualization of the vertical limb of the T capsulotomy and interportal capsulotomy, which is difficult when attempted with the hip out of traction and flexed. Our technique also helps to reduce the risk of iatrogenic cartilage injury during suture passage by increasing the distance between the femoral head and capsule leaflets, or the functional working area for capsule closure.

## Introduction

Hip arthroscopy surgery has become an increasingly common procedure in the management of intra-articular hip pathologies such as femoroacetabular impingement syndrome^1^ and hip microinstability and their sequelae such as chondral injury and labral teras. A capsulotomy typically is performed by the surgeon to facilitate manipulation of surgical instruments and visualization of the femoral head–neck junction.^2^, ^3^, ^4^ Although interportal capsulotomy is preferred by some surgeons when working in the central compartment, some surgeons prefer a T capsulotomy, which provides additional exposure when working in the peripheral compartment.^3^^,^^4^ Although the method of capsulotomy varies among surgeons, capsular closure is increasingly being recognized as a critical step of this surgery, with many studies showing better functional results when capsular closure is performed,^1^^,^^4^^,^^5^ especially in the context of hip microinstability. However, capsular closure is a time-consuming and technically challenging endeavor with a steep learning curve.^2^^,^^6^

Although hip arthroscopy has been shown to be a safe and effective procedure, iatrogenic cartilage and labral injuries are not uncommon.^6^ Typically, these injuries occur during the creation of the anterolateral portal, but these structures are vulnerable to injury during the entire surgery due to the tight working space and the nature of the arthroscopic instruments implemented.^6^ Capsular closure uses a suture passing device with a sharp tip for capsular tissue penetration that has the potential to cause iatrogenic injury, especially in inexperienced hands.^7^

In this article, we will introduce the technique of passing sutures for capsular closure under traction. This technique allows for the adequate visualization of the vertical limb of the T capsulotomy and interportal capsulotomy, which is often difficult given hip positioning in flexion. This technique also reduces the risk of potential iatrogenic injury by functionally increasing the working area for capsular closure.

## Surgical Technique (With Video Illustration)

### Patient Positioning and Portal Placement

**Fig 1:**
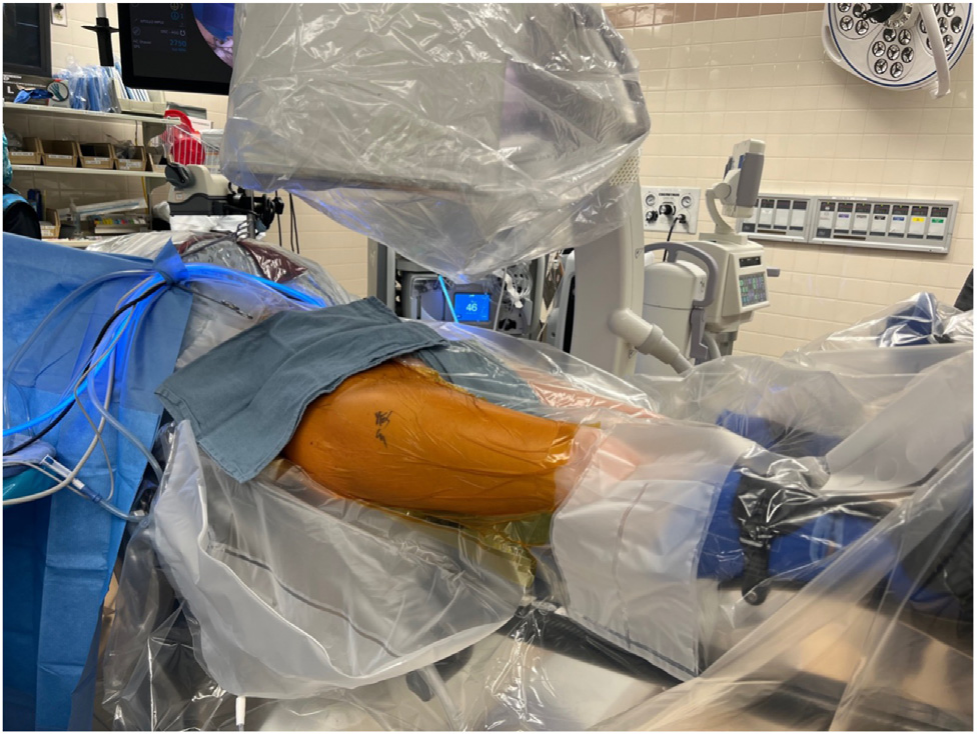
The patient is placed in a modified supine position on a traction table on a high-friction pad, in approximately 10° to 15° of Trendelenburg inclination (right hip).

**Fig 2:**
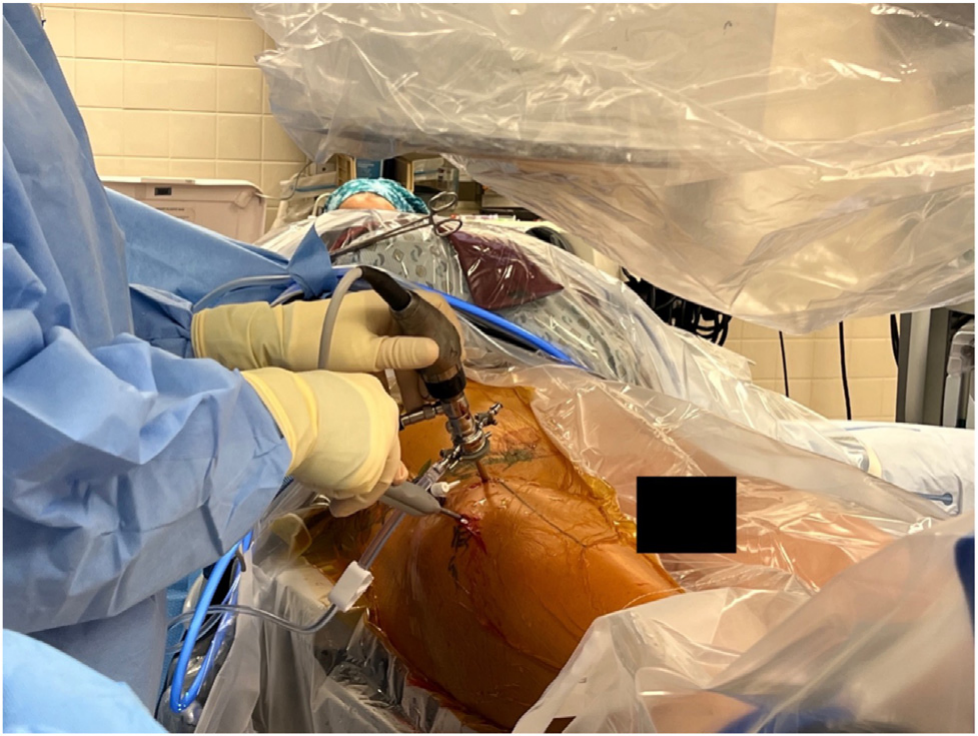
Portal placement during right hip arthroscopy. While visualization from the mid-anterior portal, a radiofrequency probe is seen in the distal anterolateral portal, which is the working portal. A plastic cannula is also seen in the anterolateral portal.

The patient is placed in a modified supine position on a traction table (Hip Distraction System; Arthrex, Naples, FL) on a high-friction pad for a postless set up (HDS-post less hip arthroscopy pad; Arthrex), in approximately 10° to 15° of Trendelenburg positioning (Fig 1). Before applying manual traction, the feet are well padded and placed in traction boots. Traction is then applied, and the operative limb is positioned in neutral adduction and rotation, and the nonoperative limb is positioned in 20° of abduction. The anterolateral portal (ALP) is created just anterior to the greater trochanter using a spinal needle with the help of fluoroscopy, followed by the creation of the mid-anterior portal (MAP) and the distal anterolateral portal (DALAP) under direct arthroscopic visualization (Fig 2).^8^

### Vertical Limb of T Capsulotomy and Central Compartment Management

**Fig 3:**
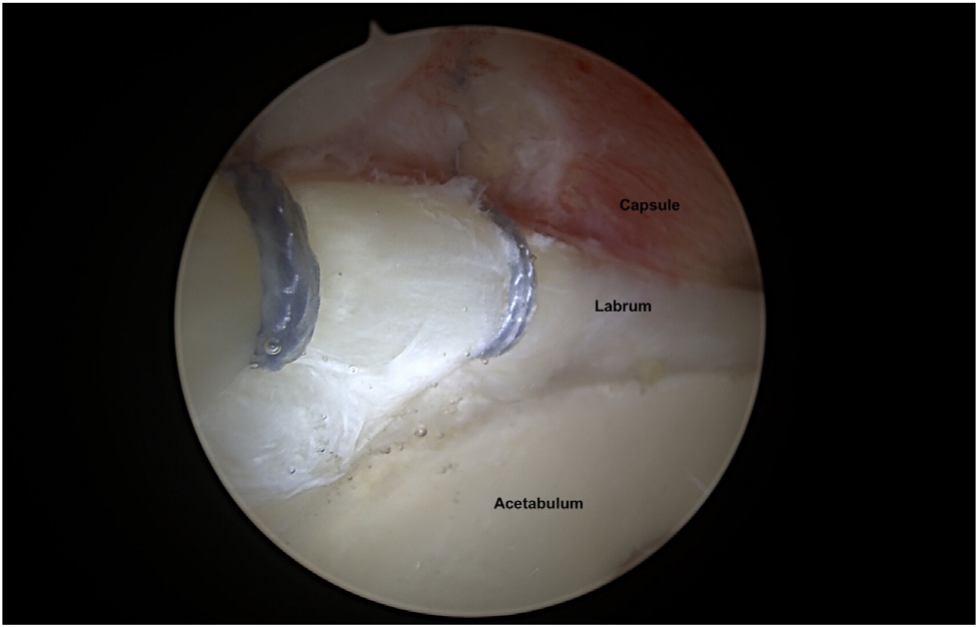
Right hip central compartment is visualized through anterolateral portal. Repaired labrum is seen.

While viewing from the MAP, the placement of the ALP is checked and confirmed to be in the 12-o’clock position and sufficiently lateral to the labrum (Video 1). An interportal capsulotomy/vertical limb of a T capsulotomy is created with an arthroscopic blade (Samurai Blade; Stryker, Kalamazoo, MI) from the MAP, leaving a cuff of tissue medially. The interportal capsulotomy is then completed by switching portals, viewing from the MAP, and working through the ALP.^3^ While viewing from the ALP, the DALAP is created. A 1.8-mm soft anchor (FiberTak; Arthrex) is placed via curved guide at the 12- to 12:30-o’clock level of the acetabulum with gentle impaction. An 8-mm plastic cannula (TransPort Canula; Stryker) is placed in the MAP and the sutures of the soft anchor are passed through the chondrolabral junction with a suture passer (Slingshot; Stryker) and the labrum is fixed with appropriate tension. The same procedure is repeated by placing a 1.8-mm soft anchor (FiberTak; Arthrex) placed at the 1:30- to 2:00-o’clock level of the acetabulum (Fig 3).

### Horizontal Limb of T Capsulotomy, Peripheral Compartment Management

**Fig 4:**
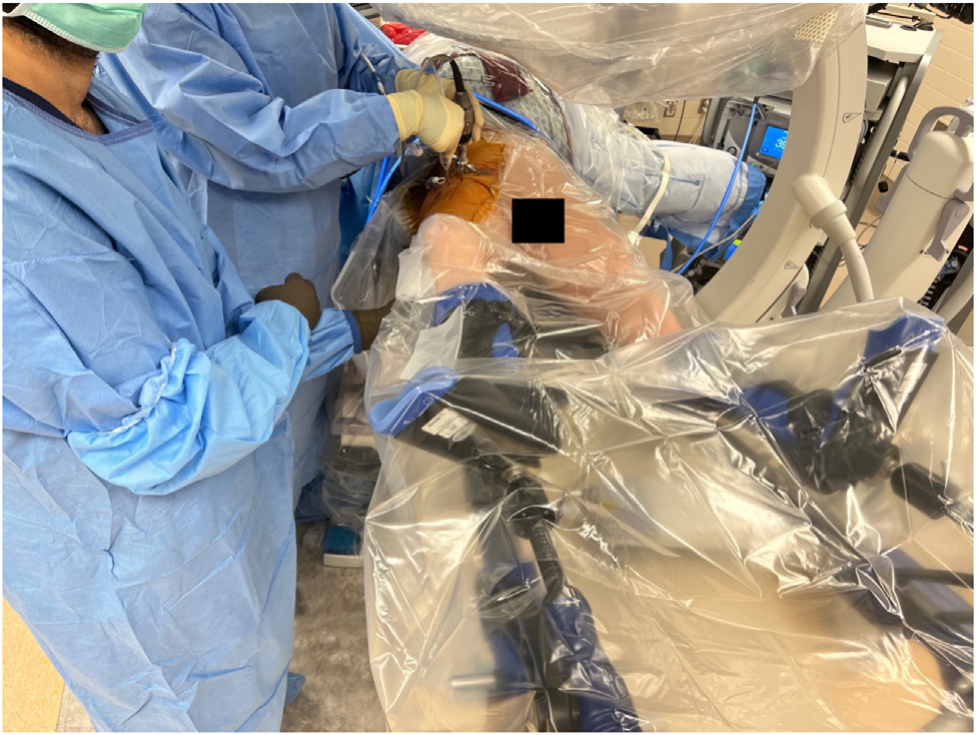
The right hip is abducted to 15° and flexed to 30°, during cam deformity resection.

**Fig 5:**
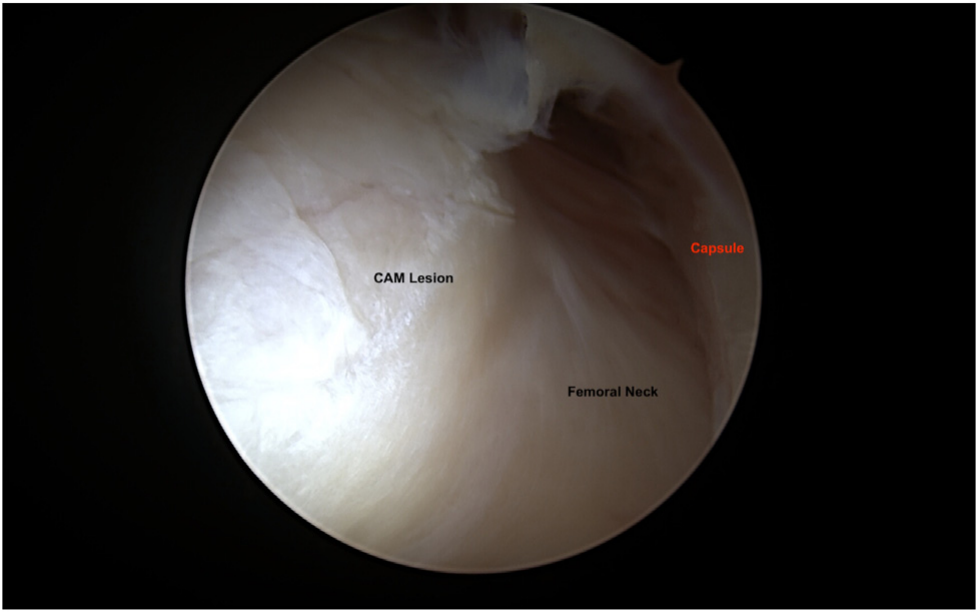
Right hip peripheral compartment is visualized through mid-anterior portal. Cam lesion is seen.

**Fig 6:**
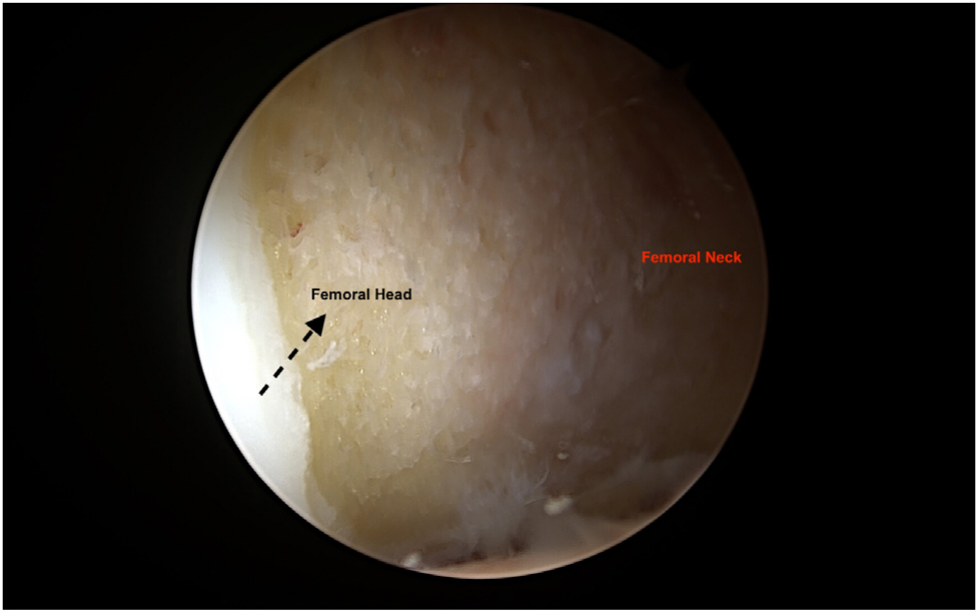
Right hip peripheral compartment is visualized through mid-anterior portal. Femoral neck is seen after femoroplasty is completed.

Once central compartment work is completed, the patient is taken out of traction and the 70° arthroscope is switched to extra-capsular region imaging from the MAP (Fig 4). With the radiofrequency probe (Arthrex) placed from DALAP, the fatty tissue located anterior to the hip capsule between the gluteus minimus and iliocapsularis muscles is debrided. Using the arthroscopic blade (Samurai Blade; Stryker) from the DALA portal, the horizontal limb of the T capsulotomy is completed by making a 2- to 3-cm horizontal capsule incision starting from the middle of the previously created vertical limb, parallel to the femoral neck. To facilitate visualization of the peripheral compartment, a #2 absorbable (VICRYL; Ethicon, Somerville, NJ) traction suture is placed on the superior (lateral) leaflet of the T capsulotomy with the help of a suture passer (Slingshot; Stryker) placed from the ALP. The hip is abducted to 15° and flexed to 30°, and the cam lesions is defined by removing periosteum with the radiofrequency probe (Arthrex) inserted from DALAP (Fig 5). Then, a 5.5-mm burr (Arthrex) is placed from the DALAP to perform the femoroplasty beginning distally and progressing proximally to the articular margin of the femoral head. After the femoroplasty is completed, the suture(s) previously placed for capsular retraction is removed (Fig 6).

### Capsular Closure

**Fig 7:**
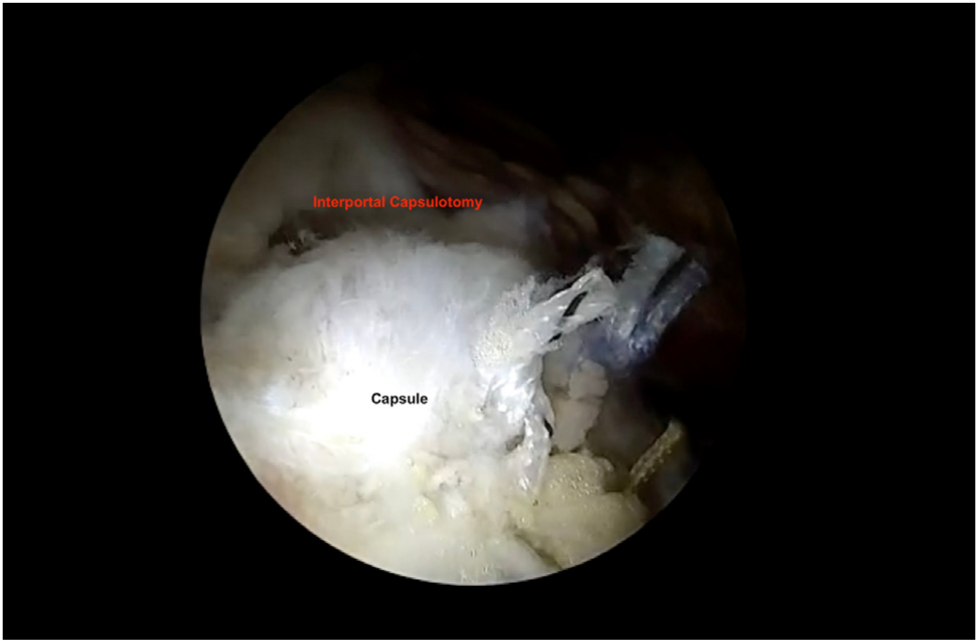
Right hip peripheral compartment is visualized through anterolateral portal. Capsule is seen after horizontal limb of T capsulotomy is repaired.

**Fig 8:**
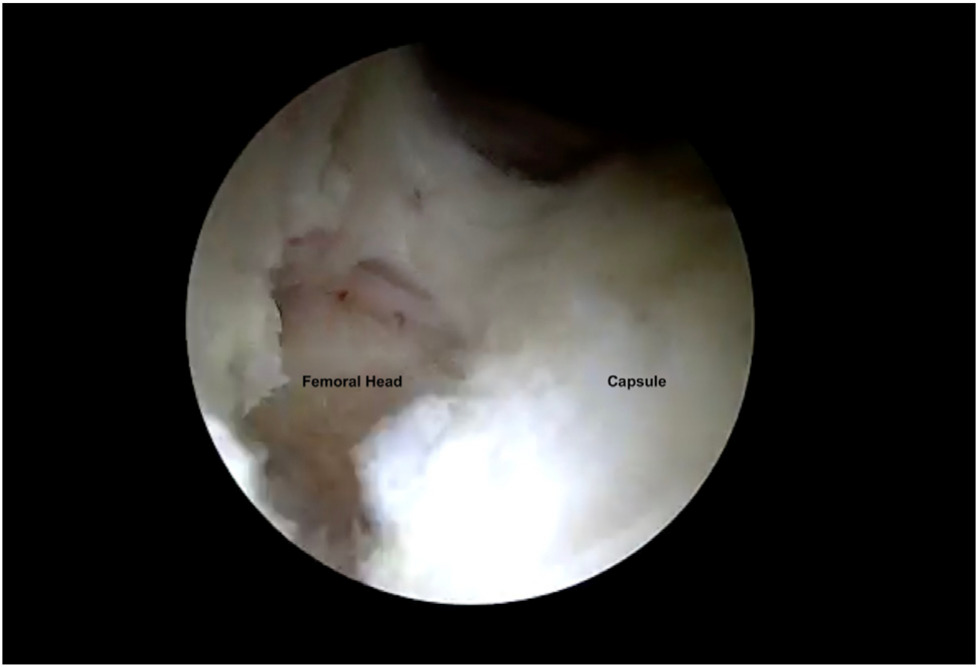
Right hip peripheral compartment is visualized through anterolateral portal. Vertical limb of T capsulotomy is seen before traction is applied.

**Fig 9:**
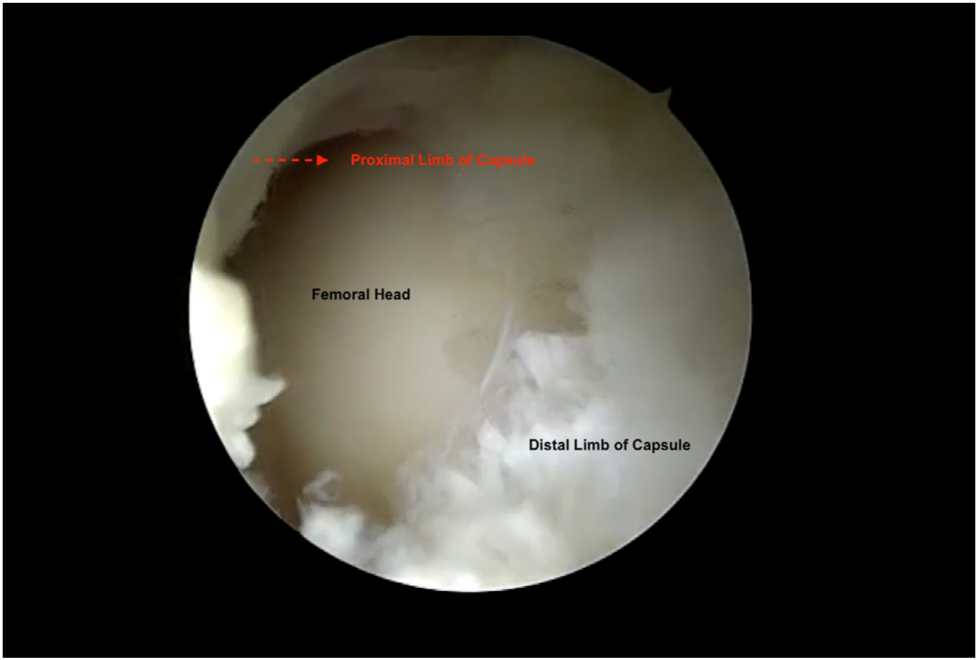
Right hip peripheral compartment is visualized through anterolateral portal. Vertical limb of T capsulotomy is seen after traction is applied.

With the hip in 15° abduction and 30° flexion, the 70° arthroscope is inserted into the MAP and an 8-mm plastic cannula (TransPort Canula; Stryker) is inserted into the ALP and DALAP. To close the horizontal limb of the T capsulotomy, three #2 nonabsorbable sutures (FiberWire; Arthrex) are placed from distal to proximal using a suture passage device (Slingshot; Stryker). During the passage of the sutures through the inferior (medial) leaflet and the superior (lateral) leaflet, the suture passer (Slingshot; Stryker) is placed from the DALAP. The same procedure is repeated for additional sutures during the closure of the horizontal limb, and they are tied sequentially (Fig 7). Before closing the vertical limb of the T capsulotomy, the hip is fully extended, and manual traction is applied (Fig 8). The traction functionally enlarges the working area, which provides easier suture passage, decreasing the risk of iatrogenic cartilage damage (Fig 9). Viewing through the MAP, three #2 nonabsorbable sutures (FiberWire; Arthrex) are placed from posterior to anterior through an 8-mm plastic cannula (TransPort Canula; Stryker). The posterior (lateral) sutures are passed through the distal limb and retrieved through the proximal limb from the ALP. The anterior (medial) sutures are passed through the distal limb from the DALA portal and retrieve it through the proximal limb from the ALP. Traction is then released, the hip is abducted 15° and flexed 30°, and the sutures are tied sequentially from anterior to posterior.

## Discussion

Although outcomes of arthroscopic treatment of hip pathology continues to improve, the most common cause of revision surgery in femoroacetabular impingement is insufficient resection of the cam deformity. One of the major contributing factors to cam under resection is inadequate intraoperative visualization.^9^ In order to increase intraoperative visualization, different capsulotomy methods have been described, with interportal and T capsulotomy being the most commonly employed techniques.^4^ Although similar postoperative functional results have been reported for both capsulotomy methods, capsule closure is an important factor in improving outcomes,^4^ especially in the context of hip microinstability. In addition, a recent systematic review showed that distractive force, total range of motion, and rotational stability of the hip increase as a result of capsular closure.^10^ Another recent biomechanical study showed that repairing the capsule provided rotational range of motion and joint translation similar to the native hip joint.^11^

Different methods have been described to close the capsule during arthroscopic treatment of the hip. In a biomechanical study comparing 4 simple sutures and 2 figure-of-eight suture methods used for closure of interportal capsulotomy, it was reported that no significant difference was found between the 2 methods.^12^ Although there is growing consensus in the literature on routine closure of the hip capsule during arthroscopy, this procedure presents challenging technical difficulties, especially for young surgeons. For this reason, self-capturing suture passing devices have been developed to facilitate the capsular closure process.^7^ However, since these devices are designed to be sharp as a nature of its function, the labrum and femoral head cartilage are at risk of iatrogenic injury due to their proximity to the working area during the capsular closure process.

In this article, we described a technique that facilitates adequate visualization of the interportal capsulotomy area. This improves the technical ease of capsule closure and also reduces the risk of iatrogenic damage (Table 1). We believe that this technique will help to facilitate ease of capsule closure and may result in more attempted, and more robust capsule closures, especially amongst inexperienced hip arthroscopists.

**Table 1:** Advantages and Limitations of Technique

Advantages
• Functionally increases the working area for capsular closure • Increases distance between femoral head and capsule reducing the risk of iatrogenic cartilage damage • Since the technique is based on tying all the capsule seams after they have been passed, the number and density of capsule closing stitches can be adjusted better.
Limitations
• Repeated traction may increase surgical time • No protection against injury to the labrum or acetabular cartilage when passing sutures through proximal leaf of capsule

## Supplementary Data

ICMJE author disclosure forms

Video 1 A case of femoroacetabular impingement, right hip. Central and peripheral compartment arthroscopy is performed first. The labrum is fixed, then femoroplasty is completed. With the hip in 15° abduction and 30° flexion, the 70° arthroscope is inserted into the mid-anterior portal (MAP) and an 8-mm plastic cannula is inserted into the anterolateral portal (ALP) and distal anterolateral portal (DALAP). To close the horizontal limb of the T capsulotomy, three #2 nonabsorbable sutures are placed from distal to proximal using a suture passage device. During the passage of the sutures through the inferior (medial) leaflet and the superior (lateral) leaflet, the suture passer is placed from the DALAP. The same procedure is repeated for additional sutures during the closure of the horizontal limb, and they are tied sequentially. Before closing the vertical limb of the T capsulotomy, the hip is fully extended, and manual traction is applied. The traction functionally enlarges the working area which provides easier suture passage, decreasing the risk of iatrogenic cartilage damage. Viewing through the MAP, three #2 nonabsorbable sutures are placed from posterior to anterior through an 8 mm plastic cannula. The posterior (lateral) sutures are passed through the distal limb and retrieved through the proximal limb from the ALP. The anterior (medial) sutures are passed through the distal limb from the DALA portal and retrieve it through the proximal limb from the ALP. Traction is then released, the hip is abducted 15° and flexed 30°, and the sutures are tied sequentially from anterior to posterior.
